# Blood transfusion‐induced posterior reversible encephalopathy syndrome presenting severe brain atrophy: A report of two cases

**DOI:** 10.1002/ccr3.5286

**Published:** 2022-01-11

**Authors:** Wataru Shiraishi

**Affiliations:** ^1^ Department of Neurology Kokura Memorial Hospital Kitakyushu City Japan; ^2^ Shiraishi Internal Medicine Clinic Nogata City Japan

**Keywords:** blood transfusion, brain atrophy, epilepsy, posterior reversible encephalopathy syndrome

## Abstract

Several cases of posterior reversible encephalopathy syndrome (PRES) after blood transfusion have been reported, but the long‐term prognosis is unknown. Here, we report two cases of blood transfusion‐associated PRES with severe brain atrophy at 1 year after onset. We report the case with a discussion of pathological mechanisms.

## Graphical abstract

This is the first report of two cases with severe cerebral atrophy after post‐transfusion PRES. Pathomechanisms are unclear, but brain hemorrhage and epileptic activity may lead to severe neuronal and glial loss in these two patients.

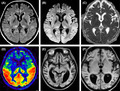

## 1 INTRODUCTION

Posterior reversible encephalopathy syndrome (PRES) is a clinical and radiological entity that presents with acute neurological symptoms, including headache, encephalopathy, seizures, and visual disturbance.^1^ In addition, various factors can trigger PRES, such as hypertension, renal failure, drugs, sepsis, and blood transfusion.^2^ The clinical course of PRES is usually self‐limiting, but the prognosis may fluctuate from a complete recovery to death due to the complications.^3^ In this case report, we describe two cases with severe cerebral atrophy at 1 year after the onset of post‐transfusion PRES, with severe residual symptoms. The long‐term prognosis of post‐transfusion PRES is unclear, and no previous reports describe a similar course to these two cases.

## 2 CASE REPORTS

In this case report, we complied with all ethical guidelines of our institution.

### 2.1 Case 1

**FIGURE 1:**
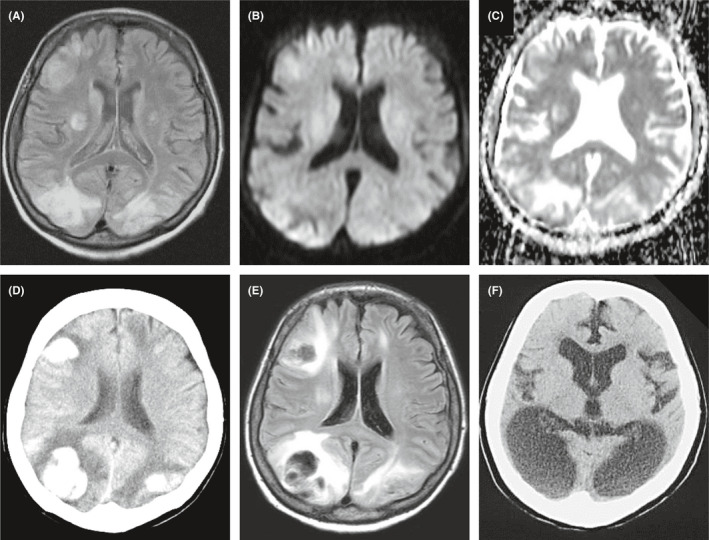
Head MRI and CT of Case 1. MRI on admission shows high‐intensity lesions in FLAIR, DWI, and ADC (A–C). Follow‐up CT and MRI show multiple intracranial hemorrhages (D, E). One‐year follow‐up CT showing occipital lobe‐dominant brain atrophy (F)

A 36‐year‐old Japanese woman was transferred to our hospital for tonic‐clonic seizure and consciousness disturbance. She maintained an unbalanced diet, eating only sweets and instant noodles. At 18 days before admission, anasarca and general fatigue appeared, and she was admitted to another hospital for severe chronic iron deficiency anemia (hemoglobin concentration, 1.4 g/dl). She reported awareness of excessive menstruation, but neglected it. An upper gastrointestinal endoscopy showed no abnormalities. She refused to undergo lower gastrointestinal endoscopy. She received 8 units during red‐blood‐cell transfusion, her hemoglobin concentration recovered to 10 g/dl, and she was discharged. The following night, the patient developed a generalized tonic‐clonic seizure and was transferred to our hospital. We performed magnetic resonance imaging (MRI) of the brain, which revealed high‐intensity lesions in fluid‐attenuated inversion recovery imaging (FLAIR), diffusion‐weighted imaging (DWI), and apparent diffusion coefficient (ADC) (Figure 1A–C). On laboratory examination, her serum glucose level was 125 g/dl (normal; 70–109 g/dl), C‐reactive protein (CRP) level was 0.04 mg/dl (normal; <0.14 mg/dl), and prothrombin time international normalized ratio (PT‐INR) was 1.35 (normal; 0.85–1.15). There was no elevation of hepatic devitalization enzymes.

Electroencephalography (EEG) showed generalized high‐voltage slow waves without epileptiform discharge. From MRI findings, we diagnosed her as PRES. However, we could not rule out encephalitis or autoimmune encephalopathy. Thus, we treated the patient with steroid pulse, antibiotics, and antiepileptic drugs (valproic acid 800 mg/day) as empiric therapy. Eventually, however she developed cerebral hemorrhage that caused hemiplegia, blindness, and mental alteration (Figure 1D,E). In addition, she had a fat‐soluble vitamin deficiency and developed night blindness due to vitamin A deficiency. Previously, we reported the patient's acute medical course.^4^ Her hemiplegia, blindness, and psychiatric symptoms have continued. She is unable to communicate or follow instructions. As a result, she has been transferred to a psychiatric hospital. At 1 year after onset, her head computed tomography showed marked atrophy of the occipital lobe (Figure 1F).

### 2.2 Case 2

**FIGURE 2:**
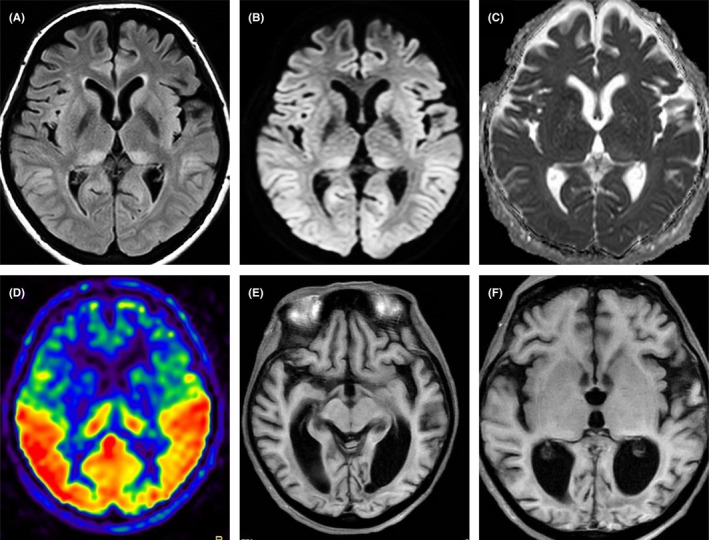
Head MRI of Case 2. MRI on admission shows high‐intensity lesions in the occipital lobe and bilateral thalamus in FLAIR and DWI (A, B). These lesions show isointense in ADC (C). ASL shows hyper‐perfusion in the occipital and thalamic lesions (D). One‐year follow‐up MRI shows occipital lobe‐dominant brain atrophy (E, F)

A 52‐year‐old Japanese woman was admitted to our hospital with consciousness disturbance and tonic‐clonic seizure. She had severe menorrhagia, but was untreated. Ten days before admission, she received 10 units during red‐blood‐cell transfusion for severe anemia (hemoglobin level, 2.2 g/dl). Brain MRI revealed hyperintensity of the bilateral thalamus and occipital lobe in FLAIR and DWI, with isointensity in ADC (Figure 2A–C). Arterial spin‐labeled imaging (ASL) showed hyper‐perfusion in the hyperintense lesions (Figure 2D). Laboratory examination revealed a serum glucose level of 98 g/dl (normal; 70–109 g/dl), a CRP level of 5.8 mg/dl (normal; <0.14 mg/dl), and prothrombin time international normalized ratio (PT‐INR) at 1.02 (normal; 0.85–1.15). Her serum liver enzymes showed mild elevation. All other blood tests, including lactic acid, pyruvic acid, and autoantibodies, were normal or negative. Cerebrospinal fluid analysis revealed normal protein and glucose levels without pleocytosis. Her EEG showed periodic sharp waves. We administered anti‐hypertensives and antiepileptic drugs (valproic acid 800 mg/day and phenobarbital 150 mg/day). Her tonic‐clonic seizure subsided and consciousness improved gradually. Follow‐up MRI after 3 weeks showed resolution of the high‐intensity lesion of the occipital lobe and thalamus. Also, ASL showed a complete recovery of hyper‐perfusion. The EEG epileptiform abnormality disappeared, but the occipital lobe‐dominant theta‐range slow‐wave activity remained. Despite laboratory data improvement, psychiatric symptoms and cortical blindness remain. She is unable to follow instructions and verbal communication is minimal. We reported the patient's acute medical course previously.^5^ She was transferred to a psychiatric hospital for the continuation of medical treatment. At 1 year after onset, she underwent head MRI, which showed marked atrophy of the brain (Figure 2E,F). We suspected hydrocephalus and administered a tap test, resulting in no recovery of her symptoms. Her cerebrospinal fluid analysis was unremarkable.

## 3 DISCUSSION

Posterior reversible encephalopathy syndrome is an acute neuroradiological manifestation, and the syndrome can involve or extend beyond the posterior cerebrum. The known risk factors of PRES are drugs, hypertension, pre‐eclampsia, renal failure, autoimmune disorders, and, in our cases, blood transfusion.^3^ Although most cases recover from PRES with appropriate management, some patients develop permanent cerebral damage and are left with residual neurological disorders. There are several reports about the poor prognostic factors of PRES. Siebert et al. reported that higher age, higher CRP level, etiology of PRES, altered coagulation, altered mental state at onset, and subarachnoid hemorrhage were related to in‐hospital death from PRES.^6^ In addition, Legriel et al. showed that the highest glycemia on admission and time to causative‐factor control were strong predictors of good outcomes in PRES patients.^7^ According to these previous reports, our two cases showed poor prognostic factors. Case 1 showed a coagulation abnormality (prolonged PT‐INR) and brain hemorrhage. Case 2 showed CRP elevation on admission. Both cases presented mental alteration and psychiatric symptoms, which remained at follow‐up. Our patients developed marked cerebral atrophy at 1 year after the onset of PRES. Brain volume reduction caused by normal aging has been reported as 0.32% per year throughout the lifetime.^8^ Our cases show a much higher degree of cerebral atrophy progression, suggesting a pathological mechanism. Brain atrophy associated with aging is correlated with diabetes mellitus, age, obesity, alcohol consumption, and white‐matter lesions.^9^ Case 1 was complicated by cerebral hemorrhage, which may be related to post‐hemorrhagic cerebral atrophy. Case 2 was found to have epileptic discharges on EEG on admission. Subsequently, the epileptic discharges disappeared, but we suspect that subclinical epilepsy persisted, which may have affected the brain atrophy, as the persistence of epileptic discharges is known to induce brain atrophy.^10^ While undiagnosed neurodegenerative diseases such as juvenile Alzheimer's disease or hereditary neurological diseases may cause the observed brain atrophy, this possibility was considered unlikely because the two cases did not show cerebral atrophy at the time of admission. Also, both cases were young women considered to be at low risk for neurodegenerative diseases. The specific cause of the cerebral atrophy in the two cases is unclear, but some cases of post‐transfusion PRES may result in such severe sequelae.

## 4 CONCLUSIONS

In this case report, we describe two cases of post‐blood‐transfusion PRES patients with severe residual symptoms and severe cerebral atrophy at 1‐year follow‐up. Pathomechanisms of these patients' cerebral atrophy are unclear. However, brain hemorrhage and epileptic activity may lead to a severe neuronal and glial loss in these two patients. An accumulation of similar cases is needed to investigate this phenomenon further.

## CONFLICTS OF INTEREST

The authors state that they have no conflicts of interest to declare.

## AUTHOR CONTRIBUTION

Wataru Shiraishi conceptualized the study and wrote, reviewed, and edited the manuscript.

## ETHICAL APPROVAL

Consent was obtained from the patients' guardians.

## CONSENT

Written informed consent was obtained from the patients' next of kin to publish this report in accordance with the journal's patient consent policy.

## Data Availability

All data included in this report are accurate to the best of our knowledge. The data are available (images and reports) upon request.
